# How artificial intelligence could improve the diagnosis and management of chronic obstructive pulmonary disease: a perspective from GOLD

**DOI:** 10.1093/ajrccm/aamag337

**Published:** 2026-06-30

**Authors:** Alvar Agusti, Marc Vila, Rosa Faner, Frederik Trinkmann, Wim Janssens, David M G Halpin, Bartolom R Celli, Jinping Zheng, Gerard Criner, Shawn D Aaron, Nicolas Roche, Leo Fabbri, MeiLan Han, Don D Sin, Fernando J Martínez, Alberto Papi, Thierry Troosters, Antonio Anzueto, Obianuju B Ozoh, Jadwiga Wedzicha, Sundeep Salvi, Dave Singh, Maria Montes de Oca, Ian Pavord, Jean Bourbeau, Claus F Vogelmeier

**Affiliations:** Cathedra Respiratory Health, University of Barcelona, Barcelona, Spain; Pulmonology Service, Respiratory Institute, Clinic Barcelona, Barcelona, Spain; Fundació Clinic per a la Recerca Biomédica, Institut d’Investigacions Biomèdiques August Pi i Sunyer (IDIBAPS), Barcelona, Spain; CIBER Enfermedades Respiratorias (CIBERES), Instituto de Salud Carlos III (ISCIII), Madrid, Spain; Cathedra Respiratory Health, University of Barcelona, Barcelona, Spain; Universitat de Vic—Universitat Central de Catalunya (UVIC-UCC), Facultat de Ciències de la Salut i el Benester (FCSB), Vic, Spain; Equip d’Assistencia Primaria Vic (EAPVIC, SLP), Vic, Spain; Cathedra Respiratory Health, University of Barcelona, Barcelona, Spain; Fundació Clinic per a la Recerca Biomédica, Institut d’Investigacions Biomèdiques August Pi i Sunyer (IDIBAPS), Barcelona, Spain; CIBER Enfermedades Respiratorias (CIBERES), Instituto de Salud Carlos III (ISCIII), Madrid, Spain; Department of Immunology, University of Barcelona, Barcelona, Spain; Department of Pneumology and Critical Care Medicine, Thoraxklinik at Heidelberg University Hospital, Translational Lung Research Center Heidelberg (TLRC), German Center for Lung Research (DZL), Heidelberg, Germany; Department of Biomedical Informatics, Center for Preventive Medicine and Digital Health, University Medical Center Mannheim, Heidelberg University, Heidelberg, Germany; Department of Chronic diseases and Metabolism KU Leuven, University Hospitals Leuven, Leuven, Belgium; University of Exeter Medical School, Faculty of Health and Life Sciences, University of Exeter, Exeter, United Kingdom; Harvard University, Boston, United States; Guangzhou Institute of Respiratory Health, First Affiliated Hospital of Guangzhou Medical University, Guangzhou, China; Lewis Katz School of Medicine at Temple University, Philadelphia, PA, United States; The Ottawa Hospital Research Institute, University of Ottawa, Ottawa, Ontario, Canada; Respiratory Medicine, Cochin Hospital, APHP Centre, Université Paris Cité and INSERM UMR1016, Cochin Institute, Paris, France; Section of Respiratory Diseases, Department of Translational Medicine, University of Ferrara, Ferrara, Italy; Division of Pulmonary and Critical Care, University of Michigan, Ann Arbor, MI, United States; Centre for Heart Lung Innovation and Division of Respirology, University of British Columbia, Vancouver, Canada; University of Massachusetts Chan Medical School, Worcester, United States; Department of Respiratory Medicine, University of Ferrara Director, Respiratory Unit, Ferrara, Italy; Cardio Respiratory Department, University Hospital S.Anna, Ferrara, Italy; Department of Rehabilitation Sciences, KU Leuven, Leuven, Belgium; University of Texas, Health San Antonio, San Antonio, TX, United States; Department of Medicine, University of Lagos College of Medicine, Lagos, Nigeria; National Heart and Lung Institute, Imperial College London, London, United Kingdom; Pulmocare Research and Education (PURE) Foundation, Pune, Symbiosis Medical College for Women and Symbiosis University Hospital and Research Centre, Symbiosis International (Deemed University), Pune, India; Division of Immunology, Immunity to Infection and Respiratory Medicine, University of Manchester, Manchester University NHS Foundation Trust and Medicines Evaluation Unit an IQVIA Business, Manchester, United Kingdom; Central University of Venezuela, Hospital Universitario de Caracas, Universidad Central de Venezuela, Centro Médico de Caracas; Respiratory Medicine Unit and Oxford Respiratory NIHR BRC, Nuffield Department of Medicine, University of Oxford, Oxford, United Kingdom; Respiratory Division, Department of Medicine, McGill University Health Centre and McGill University, Montreal, Canada; Department of Medicine, Pulmonary and Critical Care Medicine, University of Marburg, German Center for Lung Research (DZL), Marburg, Germany

## Introduction

The 2026 report of the Global Initiative for Chronic Obstructive Lung Disease (GOLD) includes a new chapter on the potential uses of artificial intelligence (AI) in chronic obstructive pulmonary disease (COPD).^1^ This perspective expands it by discussing in more detail the potential use of AI in the diagnosis and management of COPD. Of note, this is not a systematic review. Rather it is a narrative perspective based on a PubMed search using the terms “COPD OR Chronic Obstructive Pulmonary Disease” and “Artificial Intelligence.” It was written by consensus of the GOLD Science Committee members with some external AI experts invited to contribute. Table 1 presents key issues and challenges of AI for COPD diagnosis and management, and Figure 1 summarizes potential clinical benefits of AI in COPD, all of which are discussed below in more detail.

**Figure 1:**
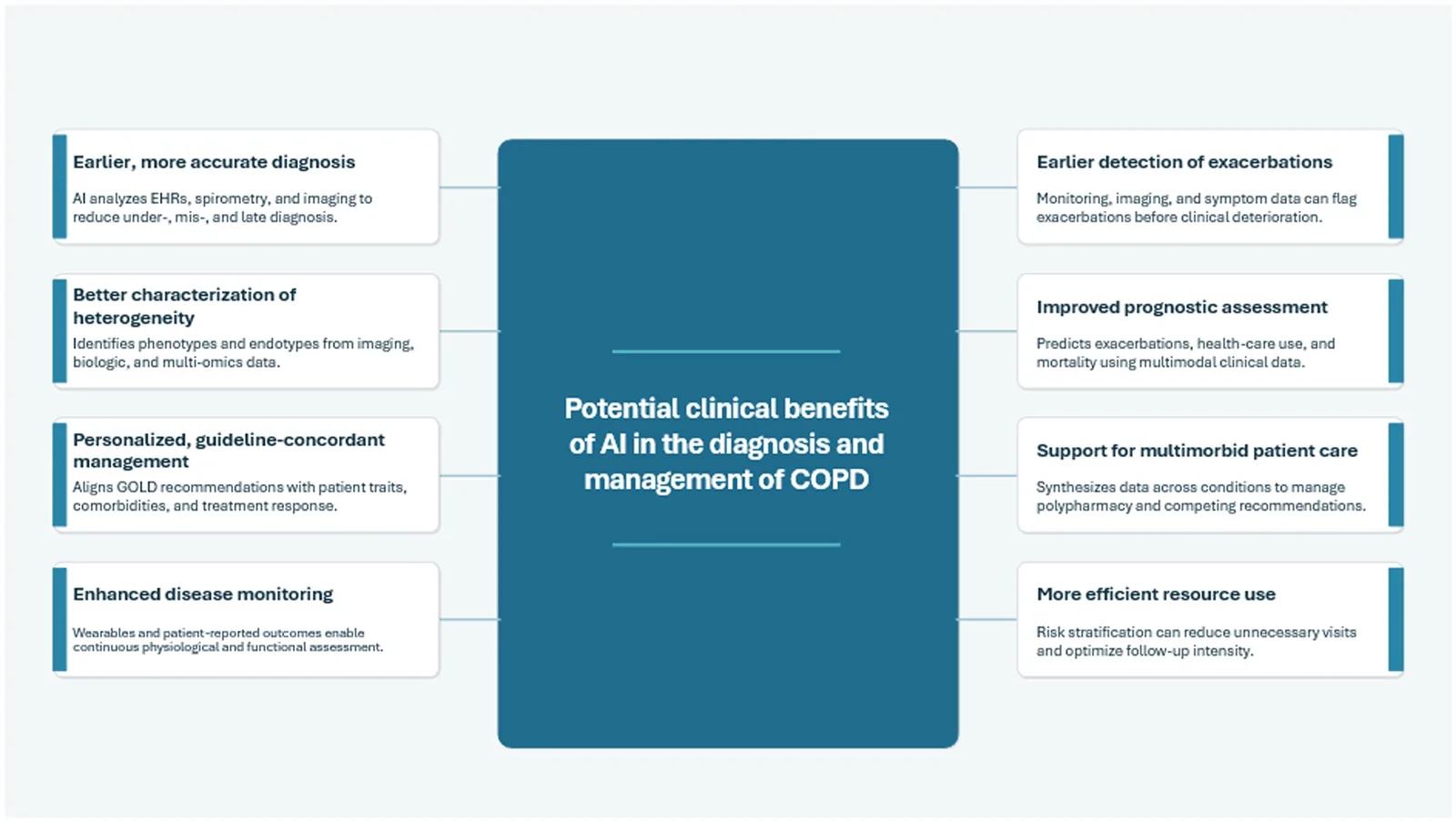
Summary of potential clinical benefits of AI in the diagnosis and management of COPD. Image created by Chat GPT on request from authors. For further explanations, see text. Abbreviations: AI, artificial intelligence; EHR, electronic health record; COPD, chronic obstructive pulmonary disease; GOLD, Global Initiative for Chronic Obstructive Lung Disease.

**Table 1:** Key issues and challenges of AI for COPD diagnosis and management.

Domain	Key issue	Brief explanation
**Evidence**	Limited prospective validation	Most AI tools for COPD management remain insufficiently validated in large, prospective, real-world cohorts.
**Outcomes**	Uncertain clinical benefit	Evidence that AI-supported interventions, particularly remote monitoring, improve hard clinical outcomes is limited.
**Generalizability**	Restricted transferability	Models developed in selected cohorts or health systems may not perform consistently across populations or settings.
**Data quality**	Dependence on high-quality data	Artificial intelligence performance relies on accurate, complete, and longitudinal clinical, imaging, and patient-reported data.
**Interpretability**	Limited transparency	Many AI models function as “black boxes,” reducing clinician trust and interpretability of recommendations.
**Guidelines**	Alignment with GOLD recommendations	Artificial intelligence outputs must be consistent with established guidelines, particularly in multimorbid patients.
**Multimorbidity**	Clinical complexity	High comorbidity burden in COPD complicates AI-based decision support and risk prediction.
**RPM implementation**	Alert fatigue and threshold setting	Defining meaningful alarms in multidimensional monitoring data is challenging and may lead to over-alerting.
**Equity**	Risk of bias	Nonrepresentative training data may reinforce existing disparities in COPD care.
**Workflow**	Integration into practice	Effective deployment requires seamless EHR integration without increasing clinician workload.

Abbreviations: AI, artificial intelligence; COPD, chronic obstructive pulmonary disease; EHR, electronic health records; GOLD, Global Initiative for Chronic Obstructive Lung Disease; RPM, remote patient monitoring.

## Artificial intelligence and COPD diagnosis

According to GOLD 2026, the diagnosis of COPD requires confirmation of persistent airflow obstruction by forced spirometry in the appropriate clinical context.^1^ However, spirometry is greatly underused, unavailable in many settings, and often misinterpreted, so underdiagnosis, misdiagnosis, and late diagnosis are key bottlenecks in COPD management. Importantly, underdiagnosis implies lack of treatment, misdiagnosis can lead to inadequate management, and late diagnosis implies that treatment is not started until COPD is already severe and less responsive.^1-3^ Artificial intelligence can help in all three scenarios.

### How can AI address COPD underdiagnosis?

Approximately 70% of COPD patients worldwide are not diagnosed.^4^^,^^5^ Improved education of the general population, policy makers, and health care professionals on the symptoms, risk factors, diagnostic criteria, and prevalence of COPD is important to reduce underdiagnosis.^1^ Artificial intelligence can also potentially help reduce COPD underdiagnosis by extracting information suggestive of COPD from electronic health records (EHRs) (eg, smoking, prematurity, repeated infections, exposure to environmental hazards, family history, symptom descriptions, treatment patterns in addition to natural language processing of radiologist reports and deep learning–based analysis of chest computed tomography [CT] images where available) that may signal undiagnosed COPD. Artificial intelligence can subsequently alert the health care provider to initiate testing for COPD in appropriate patients.^6^ Similarly, AI may facilitate cost-effective active case-finding of undiagnosed people with airway disease in the community.^7-10^ Finally, AI can spur opportunistic case finding by identifying and quantifying CT scan–associated structural lung abnormalities associated with COPD, such as emphysema or airway wall thickening in individuals included in lung cancer screening programs.^11^ For example, Tang  et al.^12^ applied deep residual networks on low-dose thoracic CTs of 2589 people who smoke in a lung cancer screening program and showed that this AI-driven approach could accurately identify individuals with COPD (based on spirometry of FEV_1_/FVC < 0.7 and FEV_1_ less than 80% of predicted) from those without COPD with an area under the curve (AUC) of 0.87. In contrast, application of traditional quantitative measures of emphysema such as mean lung density yielded an AUC of only 0.68 in the same dataset.^12^ Interestingly, in the deep-learning algorithm, the activation areas were predominantly located in the trachea and in the lung peripheries, two sites that are typically ignored in the traditional measures of lung density or airway caliber.^13^ Another example of the use of AI to predict lung function was provided by Ueda and colleagues, who applied deep-learning models to 141 734 chest x-rays and spirometry pairs of 81 902 patients across five institutions and showed that this AI-driven approach could predict FEV_1_ of patients with a Pearson correlation coefficient of 0.92 and mean root error of 0.10.^14^ Thus, with this algorithm, clinicians could use a simple chest x-ray to accurately predict patient’s FEV_1_ and institute therapy if patients have symptoms, albeit confirmation of airflow obstruction by spirometry is still recommended.^1^ Together, these studies show the power of AI methods, especially deep learning, in accurately identifying patients with COPD from CT scans or chest x-rays obtained for some other indications.

### How can AI address COPD misdiagnosis?

Between 30% and 60% of patients with physician-diagnosed COPD do not have airflow obstruction when spirometry is performed, so the patient is wrongly “labeled” as COPD based exclusively on clinical (not functional) basis.^4^^,^^15^ This can lead to inappropriate therapy and the use of unnecessary medication before a diagnostic spirometry is performed. Further, several studies have shown that AI-based quality control of spirometry is superior to human interpretation, facilitates diagnostic accuracy, and may reduce the risk of mistaken diagnoses of other clinical conditions (eg, heart failure or obesity).^16-21^ Likewise, AI can help identify potential misdiagnoses of COPD by alerting the health care provider to discrepancies between a patient’s diagnostic label of COPD and absent spirometry results, or spirometry results that do not show airflow obstruction, or inconsistent imaging findings that may support an alternative diagnosis other than COPD.^22^^,^^23^

### How can AI address late COPD diagnosis?

Generally, COPD is diagnosed (if ever) in individuals in their sixth to seventh decade of life, when the disease is advanced and relatively unresponsive to treatment.^1^^,^^24^ Diagnosis at a younger age may improve the efficiency of current therapies and prevent disease progression.^24^ As discussed above, AI can facilitate earlier COPD diagnosis by interrogating EHRs along with available imaging data to search for individuals at risk of COPD. Using data from the Framingham cohort, an individualized population-based prediction model of lung function trajectory and risk of COPD was developed using machine-learning algorithms.^25^ Similarly, an AI model was used to predict accelerated lung function decline in a prospective 4-year observational study among 13 260 individuals from the UK Royal College of General Practitioners and Surveillance Centre database.^26^ Future research is needed to explore how individual longitudinal spirometry data combined with other patient data can identify lung function trajectories and outcomes that require intervention, hence, avoiding or reducing late COPD diagnosis.^27^^,^^28^

### Artificial intelligence–based diagnostic alternatives to spirometry

Several potential AI-based alternatives to spirometry for the diagnosis of COPD are being currently investigated, but most have been tested in relatively small populations and require validation in larger cohorts.^29^^,^^30^ These include analysis of respiratory sounds^31^ and voice features,^32^ forced oscillometry,^33^ single breath nitrogen test,^34^ e-nose signals,^35^ electrocardiograms analyzed via deep learning,^36^ chest CT,^2^^,^^13^ and electrical impedance tomography.^37^ Further, AI may be able to extract novel, hidden, high-dimensional features from the flow-volume spirometry loops.^20^^,^^38^

## Artificial intelligence and COPD management

Artificial intelligence can contribute to COPD management in several complementary ways, as discussed below.

### Addressing patients′ heterogeneity using AI

#### Artificial intelligence and imaging biomarkers

Artificial intelligence–supported imaging analysis of CT chest scans is the most developed AI-based strategy currently available in COPD. Several studies showed that AI can address the structural heterogeneity of COPD. Angelini  et al. showed that unsupervised machine learning on CT scans from participants in the SPIROMICS and MESA Lung Studies identifies six emphysema subtypes.^39^ Lin et al. showed in a multicenter retrospective study of 4106 COPD patients that quantitative CT can describe the heterogeneity of alterations in airways and lung parenchyma.^40^ Finally, AI can help in the quantification of mucous plugs in the airway, a novel potential treatable trait.^41^ Artificial intelligence can estimate the clinical and physiological impact of the disease. Humphries et al., in the COPDGene and ECLIPSE cohorts, used deep-learning automation of the Fleischner grade of emphysema on CT and showed that it was associated with lung function variables and the risk of mortality.^42^ Similarly, Gawlitza et al. demonstrated that pulmonary function in COPD can be predicted from quantified CT using machine learning.^43^ Very recently, a new COPD diagnostic scheme integrating respiratory symptoms, respiratory-related quality of life, spirometry, and structural lung abnormalities on CT imaging newly classified individuals as having COPD even in the absence of spirometrically confirmed chronic airflow obstruction.^44^ These individuals had an increased risk of all-cause and respiratory-related death, frequent exacerbations, and rapid lung function decline.^44^ Additionally, AI can predict the risk of exacerbations and mortality. Gonzalez et al. showed that a deep-learning approach on CT imaging data can identify people who smoke who have COPD and predict which individuals are most likely to develop exacerbations and have the highest mortality.^45^ Likewise, a retrospective study by Feng et al. on patients with COPD found that inspiratory and expiratory high resolution CT scans can effectively predict acute exacerbations of COPD.^46^ Finally, AI can perform volumetric and pattern feature analysis of lung nodules that can avoid invasive procedures and identify patients at higher risk of lung cancer.^47^^,^^48^

#### Artificial intelligence and biologic biomarkers

Several studies have used AI to identify biologic biomarkers in COPD. Although they also require validation in larger cohorts with longitudinal data,^49^ collectively they pave the way to predict incident COPD and identify patients at higher risk of exacerbations.^50^ Additionally, by identifying biomarkers associated with disease-associated pathways, they may help identify novel therapeutic targets.

*Blood biomarkers.* Mostafaei et al. used machine learning in probes generated from small airway epithelium samples from healthy people who smoke, healthy people who do not smoke, and people with COPD who smoke and identified 44 candidate genes, 17 of which have not been previously found in COPD.^51^ Similarly, Matsumura et al. used machine learning to identify 15 novel biomarker genes that distinguished between people who smoke and COPD patients and converted gene expression levels to a numerical index, the so-called potential risk factor index, that can quantitatively reflect the risk continuum across smoking and COPD.^52^ Finally, Olvera et al. identified multilayer omics communities of patients with different underlying biological pathways (endotypes) but similar clinical presentation (phenotypes).^53^

*Urine biomarkers.* Using artificial neural network Yousuf et al. identified a panel of biomarkers differentially expressed in stable and exacerbation state and predicted exacerbations before symptoms occur.^54^

*Vocal biomarkers.* Vocal biomarkers are emerging as promising digital tools for COPD detection and monitoring, utilizing machine-learning analysis of speech and voice characteristics to assess disease presence, severity, and exacerbation status using smartphone recordings.^55^

#### Foundation models

Foundation models are large-scale AI models trained on massive, diverse datasets that can integrate imaging, text, and structured clinical and laboratory data.^56^ However, many foundational models are generic by design and may not be organ-, modality-, or disease-specific. While they can perform multiple tasks, early evidence suggests that generalist models do not always outperform specialized approaches for specific clinical tasks (eg, disease diagnosis or risk prediction in COPD).

### Artificial intelligence to facilitate the use of disease guidelines

To be implemented in clinical practice, disease guidelines need to be, first, memorized by practicing physicians, who should then use them wisely in each patient in their clinical practice. Since many COPD patients suffer from other concomitant diseases (ie, multimorbidity), for which other guidelines exist and for which the same process of memorization and wise implementation is required,^57^ AI can be of great help for the management of multimorbid COPD patients. For instance, AI systems embedded in EHR systems could (1) summarize the current clinical status of a multimorbid patient who is cared for by different specialties and consult the appropriate individual guidelines to suggest best treatments; (2) alert the physicians of potential discrepancies vs guideline recommendations in the management of that specific patient (eg, polypharmacy, or inappropriate treatment of some component(s) of the multimorbidity present); (3) recommend preventive health measures, vaccines, or other general health care interventions (colonoscopy, mammograms, etc.); and (4) record the conversation between the patient and the doctor, facilitating a more empathic interview, at the end of which AI can produce a summary of the visit that, after being approved by the doctor, can be stored in the EHR and forwarded to the patient using a comprehensible (ie, nontechnical) language. Of note, this is already in use in some centers and practices. Finally, it could be argued that written guidelines may no longer be necessary in the era of AI. It is author's opinion that this is wrong since guidelines are written by “human consensus based on best available evidence” and, as such, they represent an invaluable source of data for AI. Needless to say, clinical judgement remains key when deciding whether and how these recommendations should be implemented in a specific clinical situation. The role of AI in the treatment of COPD (such as selection of therapeutic drugs, personalized treatment recommendations, prediction of treatment outcomes, and pharmacoeconomic forecasting) requires further investigation.

### Remote patient monitoring

Current wearable sensor technologies allow transmission of patient-reported outcomes and physiological and functional data, such as heart rate, weight, blood pressure, oxygen saturation, physical activity, and/or lung function (peak flow, microspirometers, impulse oscillometry), directly to health care professionals through web-based or phone-based data entry.^58^^,^^59^ Artificial intelligence can integrate these different measurements and propose a comprehensive summary of a patient’s health to the clinician after thresholds and alarms had been carefully determined. Importantly, it is also necessary to define who, how, and when to respond to these alarms.^60^ Although conceptually appealing and technically feasible, there is limited evidence that remote patient monitoring (RPM) in COPD impacts relevant clinical outcomes.^61^ However, some potential applications of AI to RPM in COPD patients might include monitoring adherence and appropriate use of inhaled medication and early detection of exacerbations allowing prompt intervention.^60^^,^^62^^,^^63^

### The patient perspective

Artificial intelligence can facilitate the contact of an individual with the health care system.^64^ For instance, a trained chatbot or avatar can answer questions patients may have in relation to the management of their COPD (or health at large) and make specific recommendations.^65^ Alternatively, the chatbot/avatar can adopt a more proactive approach triggered by RPM and counsel patients. Such AI-supported approaches can potentially bring health care closer to the patient and avoid unnecessary consultations while supporting effective self-management.^64^

### Artificial intelligence and prognosis in COPD

A recent systematic review and meta-analysis concluded that there is still limited evidence that AI models demonstrate superiority to preexisting disease severity scores in COPD.^66^ Yet many studies have highlighted its potential. Young et al. developed a machine-learning tool called Subtype and Stage Inference (SuStaIn) based on cross-sectional CT imaging markers to identify different trajectories of disease progression and identified two distinct patterns, likely representing different endotypes.^67^ The authors found a Tissue→Airway subtype (70%) in which small airway dysfunction and emphysema precede large airway wall abnormalities, and an Airway→Tissue subtype (30%), where large airway wall abnormalities precede emphysema and small airway dysfunction.^67^ Importantly, SuStaIn identified 30% of people who smoke with normal lung function at baseline who were 2.5 times more likely to meet COPD diagnostic criteria at follow-up, suggesting early imaging changes are predictive of eventual development of COPD.^67^ Shmatko et al. used AI to learn the grammar, contextual logic of language, and baseline health information in UK Biobank participants and showed its capacity to infer different patterns of multidisease progression.^68^ Zeng et al. developed a machine-learning model to predict severe exacerbations of COPD with an AUC receiver operating characteristic curve of 0.87.^69^ Wu et al.^70^ developed a prediction system using AI and a wearable device, lifestyle data, environmental factors (temperature, humidity, and fine particulate matter collected using a home air quality–sensing device) and patient symptoms for the early detection of exacerbations of COPD in the upcoming 7 days. The model achieved an accuracy of 92.1%, a sensitivity of 94%, and a specificity of 90.4%. The most important variables included in the model were daily steps walked, stairs climbed, and daily distance moved. Moslemi et al. showed that quantitative CT and machine learning in combination with other clinical measures lead to greater prediction accuracy of subsequent health services use (emergency room visits and hospitalizations) than conventional measurements alone.^71^ Fernandez-Granero et al. developed a system composed of electronic stethoscopes and computerized analyses of respiratory sounds with machine-learning techniques for the remote early detection of exacerbations of COPD.^72^ In a 6-month pilot study, 75.8% exacerbations were early detected with an average of 5 ± 2 days in advance at medical attention.^72^ Yuan  et al. studied patients included in the COPDGene study and showed that CT measurements may help stratify patients along the continuum of COPD phenotypes with individual potential to predict lung function decline, future exacerbation, and mortality.^73^ Yun et al. used a convolutional neural network model to extract deep radiomics features (ie, quantitative metrics) from chest CT in 344 patients from the Korean Obstructive Lung Disease cohort, with an external validation in 102 patients in Malaysia, and showed that radiomics can predict mortality in COPD patients.^74^ Finally Cosentino et al. showed that a deep convolutional neural network can predict survival and exacerbation events from raw spirograms.^75^

## Conclusions

The potential of AI in COPD is just emerging.^76-78^ Artificial intelligence in COPD can revolutionize the diagnosis of the disease; the characterization of patient heterogeneity; the deployment of precise and personalized management, including better identification and care of multimorbid diseases; the correct application of multiple existing guidelines; and the development of better prognostic tools (Figure 2). Yet, before being implemented in clinical practice, AI applications should be independently validated and adapted for local contexts using locally relevant data in representative clinical settings that show true effectiveness and clinical impact compared to current standards. Likewise, they should be deployable in resource-limited settings with minimal technical requirements as much as possible. In any case, exciting new times are here by combining AI with other technologies and strategies directed toward the prevention, diagnosis, and optimal care of people with COPD (and many other human diseases too).

**Figure 2:**
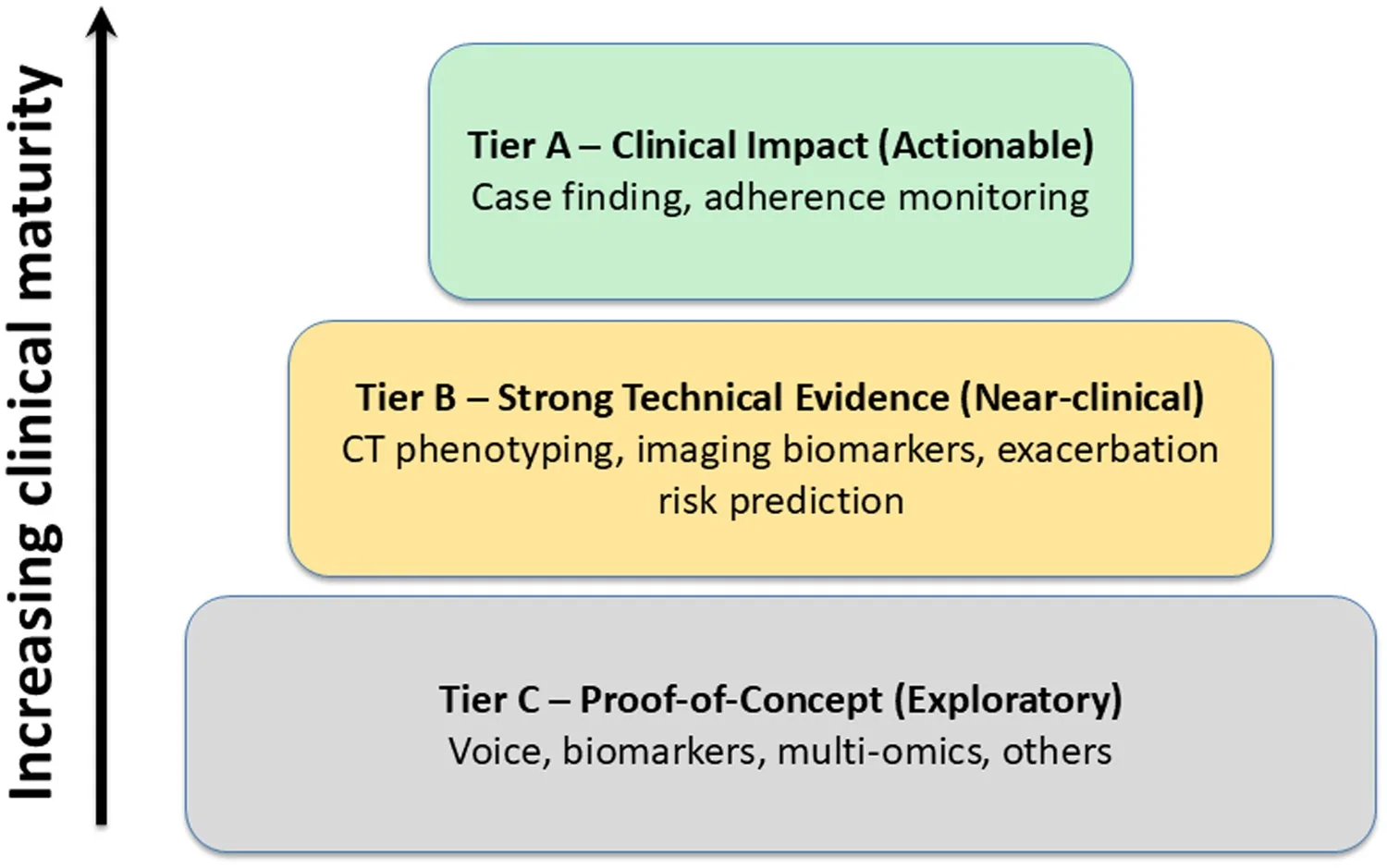
Artificial intelligence in chronic obstructive pulmonary disease: from concept to clinical impact. Abbreviation: CT, computed tomography.

## Supplementary Material

aamag337_Supplementary_Data
