# Supplementary material for: How artificial intelligence could improve the diagnosis and management of chronic obstructive pulmonary disease: a perspective from GOLD
Source: Am J Respir Crit Care Med. 2026 Jun 30;212(11):2736–44. doi: 10.1093/ajrccm/aamag337 (PMC13721132; doi:10.1093/ajrccm/aamag337)
Supplement: Supplementary file 1 [file aamag337_supplementary_data.zip › coi_disclosure OCT AI Blu ap.pdf]

ICMJE DISCLOSURE FORM

Date: 15 Oct 2025
Your Name: Alberto Papi
Manuscript Title: ARTIFICIAL INTELLIGENCE IN COPD: A VIEW FROM THE GOLD SCIENCE COMMITTEE
Manuscript number (if known): NA

In the interest of transparency, we ask you to disclose all relationships/activities/interests listed below that are related to the content of your manuscript. "Related" means any relation with for-profit or not-for-profit third parties whose interests may be affected by the content of the manuscript. Disclosure represents a commitment to transparency and does not necessarily indicate a bias. If you are in doubt about whether to list a relationship/activity/interest, it is preferable that you do so.

The following questions apply to the author's relationships/activities/interests as they relate to the current manuscript only.

The author's relationships/activities/interests should be defined broadly. For example, if your manuscript pertains to the epidemiology of hypertension, you should declare all relationships with manufacturers of antihypertensive medication, even if that medication is not mentioned in the manuscript.

In item #1 below, report all support for the work reported in this manuscript without time limit. For all other items, the time frame for disclosure is the past 36 months.

		**Name all entities with whom you have this relationship or indicate none (add rows as needed)**	**Specifications/Comments (e.g., if payments were made to you or to your institution)**
**Time frame: Since the initial planning of the work**			
1	All support for the present manuscript (e.g., funding, provision of study materials, medical writing, article processing charges, etc.) **No time limit for this item.**		
**Time frame: past 36 months**			
2	Grants or contracts from any entity (if not indicated in item #1 above).	AstraZeneca	Payments to institution
		Chiesi	Payments to institution
		GlaxoSmithKline	Payments to institution
		Sanofi	Payments to institution
3	Royalties or licenses	____None	
4	Consulting fees	AstraZeneca	Consultancy fees
		Avillion	Consultancy fees

		Chiesi	Consultancy fees
		GlaxoSmithKline	Consultancy fees
		Moderna	Consultancy fees
		Regeneron	Consultancy fees
		Roche	Consultancy fees
		Sanofi	Consultancy fees
		Zambon	Consultancy fees
		Zentiva	Consultancy fees
5	Payment or honoraria for lectures, presentations, speakers bureaus, manuscript writing or educational events	AstraZeneca	Payment or honoraria for lectures
		Avillion	Payment or honoraria for lectures
		Chiesi	Payment or honoraria for lectures
		Regeneron	Payment or honoraria for lectures
		Moderna	Payment or honoraria for lectures
		GlaxoSmithKline	Payment or honoraria for lectures
		Roche	Payment or honoraria for lectures
		Sanofi	Payment or honoraria for lectures
		Zambon	Payment or honoraria for lectures
6	Payment for expert testimony	____None	
7	Support for attending meetings and/or travel	____None	
8	Patents planned, issued or pending	____None	
9	Participation on a Data Safety Monitoring Board or Advisory Board	____None	
10	Leadership or fiduciary role in other board, society, committee or advocacy group, paid or unpaid	____None	
11	Stock or stock options	____None	
12	Receipt of equipment, materials, drugs, medical writing, gifts or other services	____None	
13	Other financial or non-financial interests	____None	

Please place an "X" next to the following statement to indicate your agreement:

_X_ I certify that I have answered every question and have not altered the wording of any of the questions on this form.
